# Dermoid cyst with secretion of CA 19-9 detected by ^18^F-FDG PET/CT: A case report

**DOI:** 10.1097/MD.0000000000018988

**Published:** 2020-03-06

**Authors:** Sheng-Che Lin, Ruoh-Fang Yen, Yen-Kung Chen

**Affiliations:** a Department of Nuclear Medicine and PET Center, Shin Kong Wu Ho-Su Memorial Hospital; b School of Medicine, Fu Jen Catholic University; c Department of Nuclear Medicine, National Taiwan University Hospital and National Taiwan University College of Medicine, Taipei, Taiwan.

**Keywords:** ^18^F-FDG PET/CT, dermoid cyst, CA19-9

## Abstract

**Introduction:**

Carbohydrate antigen 19-9 (CA 19-9) is a tumor glycolipid, frequently elevated in the serum of patients due to malignancies from gastrointestinal organs; in particular, the pancreas. This carbohydrate antigen is also expressed in benign diseases.

**Patient concerns:**

A case of a 27-year-old female who has an unknown origin CA 19-9 elevation for 2 years.

**Diagnosis:**

After the left ovarian cystectomy and microscopic examination, the final diagnosis is a dermoid cyst. The dermoid cyst shows increased ^18^F-fluorodeoxyglucose (^18^F-FDG) uptake in the ^18^F-FDG positron emission tomography (PET)/computed tomography (CT) study.

**Intervention and outcomes:**

The laparoscopic oophorocystectomy was performed. It was observed that the patient's CA 19-9 level returned to normal after the surgery 6 months later. This showed that the dermoid cyst was responsible for the abnormal CA 19-9 level.

**Conclusion:**

In this case, we can learn that the ^18^F-FDG PET/CT scan has potential use in patients with unknown origin of elevation CA 19-9.

## 1 Introduction

A dermoid cyst, also known as mature teratoma, is a common benign tumor of the ovary. It accounts for 10% to 20% of all ovarian neoplasms and is composed of a well-differentiated derivation of three germ cell layers (endoderm, mesoderm, and ectoderm). Dermoid cysts are asymptomatic in most cases and are benign over 98% cases.^[1–3]^ In our report, a dermoid cyst with secretion of carbohydrate antigen 19-9 (CA 19-9) is detected by ^18^F-fluorodeoxyglucose (^18^F-FDG) positron emission tomography (PET)/computed tomography (CT).

## 2 Clinical course

A 27-year-old female was referred to our PET center for a whole-body ^18^F-FDG PET/CT study. She had been noted to have persistently high-serum CA 19-9 up to 3498 U/mL for 2 years before this PET study in a health examination. Gastrointestinal system disease had been ruled out by endoscopy, abdominal ultrasound, CT, and magnetic resonance imaging (MRI) with negative results.

**Figure 1:**
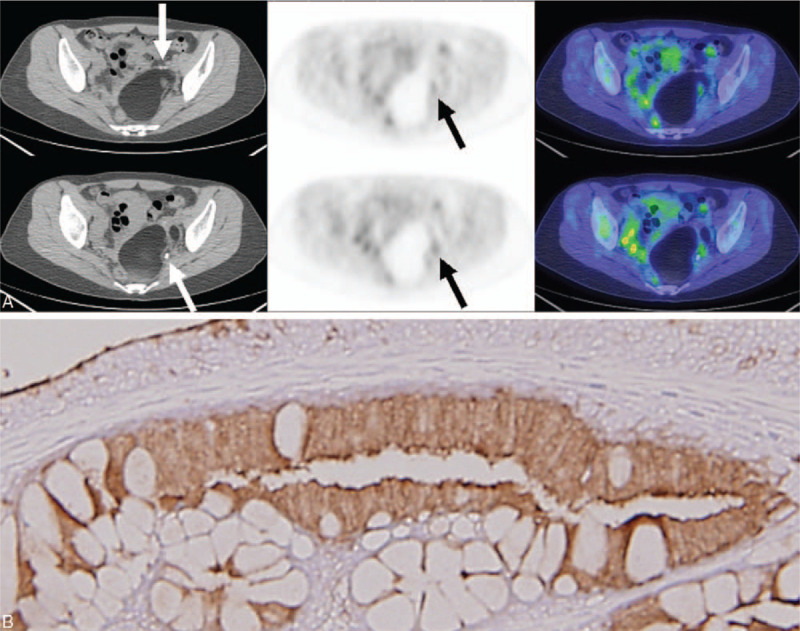
^18^F-FDG PET/CT of the large pelvic tumor (A), and diamino bezidin staining. Original magnification 200× (B). (A) CT (left), PET (middle), and fused PET/CT (right) images demonstrating a large tumor with fatty component, greasy fluid component, calcification (white arrows), and soft tissue component with ^18^F-FDG uptake in the tumor cavity (black arrows). (B) Diffuse CA 19-9 expression in the epithelium. ^18^F-FDG = ^18^F-fluorodeoxyglucose, CA 19-9 = carbohydrate antigen 19-9, PET/CT = positron emission tomography/computed tomography.

PET/CT imaging was performed 60 minutes after intravenous injection of 10 mCi ^18^F-FDG. The resulting images revealed a well-defined large tumor with fatty component, greasy fluid component, calcification of bony fragment or tooth, and soft tissue component in her left pelvic cavity. Mild ^18^F-FDG uptake was observed in the areas containing soft tissue component (Fig. 1A). Based on the PET/CT findings, the left ovarian dermoid cyst was diagnosed.

## 3 Interventions

The laparoscopic oophorocystectomy was performed. A 9.2 × 6.1 × 6.2 cm cystic lesion with smooth external surface was removed. This cyst was intact and found to have presence of hair, yellowish friable material and focal calcification and was confirmed, via microscope, to be a dermoid cyst composed of squamous epithelium, skin appendages, gastrointestinal, and respiratory tract epithelium. By immunohistochemistry, strong CA 19-9 staining was demonstrated in the epithelium (Fig. 1B).

## 4 Outcomes

It was observed that the patient's CA 19-9 level returned to normal (11.2 U/mL) 6 months after the operation. Before operation, the CA 19-9 level is 3498.3 U/mL and the CEA level is 0.47 ng/mL. This showed that the dermoid cyst was responsible for the abnormal CA-19-9 level. Informed written consent was obtained from the patient for publication of this case report and accompanying images.

## 5 Discussion

CA 19-9 is a tumor glycolipid, frequently elevated in the serum of patients due to malignancies from gastrointestinal organs; in particular, the pancreas. CA 19-9 was identified by a murine monoclonal antibody against a colorectal carcinoma epithelial cell. This carbohydrate antigen is also expressed in benign diseases, as well as pancreatic or biliary ductal epithelial cancers.^[1–3]^ However, elevated serum CA 19-9 has also been observed for some patients with either benign or malignant dermoid cysts.^[4–9]^ It has also been reported that CA 19-9 staining is prominently noted around the apical cytoplasma of the epithelial lining. This phenomenon has led Atabekoglu et al to suggest that CA 19-9 is secreted into the cystic cavity. Inflammation or rupture of dermoid cyst, or direct serum excretion via epithelial surface into the bloodstream may therefore result in the elevation of serum CA 19-9.^[7]^ As a consequence, CA 19-9 is suited for being a marker for surveying recurrence. Manni Wang report a case shows elevation of CA19-9 (1329.7 IU/mL) and FDG PET/CT positive finding. The pathological diagnosis was left ovarian cancer, an immature teratoma grade 1.^[9]^

The routine serum CA 19-9 level testing has no utility as a screening tool in asymptomatic patients. Even among patients with symptoms suspicious for pancreatic cancer, elevated CA 19-9 is a poor predictor of pancreatic cancer with a predictive value of 0.5% to 0.9%.^[10]^

We suspect that the increased ^18^F-FDG uptake at the soft tissue component of the dermoid cyst may be attributed to the glucose utilization as the energy source for the functionally active secreting epithelium. The ^18^F-FDG PET/CT scan has potential use in patients with unknown origin of elevation CA19-9.

## Author contributions

**Data curation:** ShengChe Lin.

**Investigation:** ShengChe Lin, Ruoh-Fang Yen.

**Methodology:** Yen-Kung Chen.

**Writing – original draft:** ShengChe Lin.

**Writing – review & editing:** Yen-Kung Chen.
